# The “pressure” of being an adolescent in the West Bank, Palestine: prevalence of prehypertension and hypertension

**DOI:** 10.3389/fpubh.2025.1620629

**Published:** 2025-08-20

**Authors:** Dina Hamideh, Hamzeh Al Zabadi, Noe C. Crespo, John Alcaraz, Mariam Mansour, Marcos Real, Baseel Hamideh, Nargis Ahmadi, Lamees Mahmoud, David Strong

**Affiliations:** ^1^Herbert Wertheim School of Public Health, University of California San Diego, La Jolla, CA, United States; ^2^School of Public Health, San Diego State University, San Diego, CA, United States; ^3^Public Health Department, Faculty of Medicine and Health Sciences, An-Najah National University, Nablus, Palestine

**Keywords:** cardiovascualr disease, Middle East North Africa (MENA), Palestinian adolescents, systolic blood pressure, refugee

## Abstract

**Introduction:**

Over 27% of adults in Palestine are reported to have hypertension, and there is limited knowledge about hypertension rates among Palestinian adolescents residing in the West Bank, Palestine. Prolonged exposure to forced displacement has adverse impacts on health, including the risk of hypertension. This study assessed the relationship between refugee status, prehypertension, and hypertension among Palestinian adolescents residing in the West Bank, Palestine.

**Methods:**

This cross-sectional study was conducted among Palestinian adolescents residing in refugee camps, villages, and cities across the West Bank. A random sample of adolescents was recruited from schools from November 2022 to January 2023. Anthropometric measurements, including blood pressure (mmHg), heart rate (BPM), body fat (%), height (cm), and body weight (kg), and survey questionnaires, were collected. Ordinal regression models were used to assess the relationship between refugee status, prehypertension, and hypertension after adjusting for age. In addition, the interaction between refugee status and sex and its relationship to the prevalence of prehypertension and hypertension were examined.

**Results:**

A total of 706 Palestinian adolescents [refugees = 377 (39.4% female individuals and 60.6% male individuals), and non-refugees = 329 (39.9% female individuals and 60.1% male individuals)] aged between 13 and 17 years (median = 14 years) were enrolled. The prevalence of prehypertension and hypertension in the sample was 26 and 14%, respectively. The prevalence of prehypertension [refugees = 25.5% vs. non-refugees = 27.0%] and hypertension [refugees = 12.7% vs. non-refugees = 16.0%] was higher among non-refugees. There was no association between refugee status and hypertension categories in either the unadjusted models or models adjusted for age [adjusted odds ratio (AOR) 0.88; 95% CI = 0.65–1.20]. The findings from the regression models revealed an association between refugee status, prehypertension, and hypertension among female and male participants. Among female participants, refugees had 1.75 [95%CI = 1.04–2.95] times higher odds of elevated blood pressure than non-refugees, after adjusting for age. In contrast, male refugees had 40% lower odds [AOR = 0.6; 95% CI = 0.41–0.88] of elevated blood pressure than their non-refugee counterparts.

**Conclusion:**

The prevalence of prehypertension and hypertension was high in this participant sample, highlighting a significant public health concern. Inverse relationships between blood pressure and refugee status were observed across sexes. Future studies should assess the impact of refugee and non-refugee experiences and sex differences on cardiovascular health disparities among Palestinian adolescents.

## Highlights

- *New Findings from a Clinical Perspective*: This study found that Palestinian adolescents in the West Bank had higher rates of prehypertension (26%) and hypertension (14%) compared to the global average, with sex differences in hypertension levels based on refugee status. This finding highlights a critical public health issue and underscores the need for early detection and behavioral interventions to address hypertension and cardiovascular disease (CVD) risk in this vulnerable population.
- *Clinical Implications*: The high prevalence of prehypertension and hypertension in this sample suggest that healthcare providers should prioritize early screening and risk assessments for adolescents in the West Bank, Palestine, while also considering the impact of lifestyle factors, sex, and refugee status on blood pressure.
- *Future Research Directions*: Future studies should assess the influence of lifestyle, psychosocial factors, and refugee status on adolescent blood pressure, with a focus on sex-based differences. This approach will help tailor interventions more effectively and address the specific needs of male and female adolescents in both refugee and non-refugee contexts.

## Introduction

The long-term and intergenerational health effects of forced displacement on youth include the risk of developing chronic health conditions such as diabetes, hypertension, and cardiovascular disease (CVD) (1–3). Vulnerable and exiled populations have the highest non-communicable disease burden, particularly in the Middle East and North African (MENA) region (4). The MENA region has the second-highest age-standardized prevalence rate of adult CVD mortality, with hypertension identified as one of the prominent risk factors (5). Studies have shown that adult refugee populations in the MENA region have higher odds of hypertension than non-refugee populations (6). In Palestine, 38% of deaths are due to CVD, yet refugee-specific data are limited (7).

Forced displacement has resulted in severe long-term physical and mental health burdens, particularly among Palestinian adolescent refugees within Palestine (8). During the Nakba—the Catastrophe—more than 6 million Palestinians were forcibly displaced, resulting in the world’s largest refugee population, with many residing in refugee camps in the West Bank and Gaza regions of Palestine, as well as Syria, Jordan, and Lebanon (9, 10). The West Bank is home to 846,465 of 2.5 million refugees in Palestine, with current adolescents representing the fifth generation of refugees (11). Both Palestinian refugees (i.e., those exiled following the 1948 and 1967 events) and their non-refugee counterparts (i.e., Palestinians residing in the West Bank and Gaza who were not displaced during the 1948 and 1967 conflicts) endure occupation, characterized by psychological and physical violence, restricted movement, and displacement (12). Moreover, living in overcrowded, low-resourced, and unsanitary refugee camps exacerbates chronic stress in Palestinian refugees (12). This stress often leads to the development of psychological and behavioral symptoms of depression and anxiety, which increase the risk of hypertension (13). Chronic stress can increase blood pressure and alter the neuroendocrine response to stress, raising basal blood pressure over time (14).

Hypertension among adolescents is a global health concern, with female adolescents at a higher risk of developing the condition (14–16). Studies suggest sex-based variations in psychosocial and physical responses to stress, violence, and trauma between male and female individuals, which are linked to mental well-being and physiological outcomes (17–19). Gettler et al. (20) found that male refugees had lower chronic inflammation than female refugees. Chronic inflammation affects long-term trajectories for CVD (21). However, limited research has examined the relationship between sex differences, refugee status, and hypertension in adolescents, including Palestinian refugee adolescents.

Early stages of high blood pressure, known as prehypertension, in youth frequently progress to adult hypertension (22, 23). Early detection and intervention for high blood pressure are crucial, as this condition influences cardiovascular morbidity and mortality in adulthood, as well as health outcomes such as diabetes and dyslipidemia (24, 25). The global average prevalence of hypertension among adolescents is 4%, with an alarming average of 12.6% in Arab countries (26–28). Recent studies suggest that Palestine is among these Arab countries experiencing an increasing rate of high blood pressure, ranging from 26.3 to 59.3% among Palestinian youth (i.e., ages 9–12 and 16–17) residing in the West Bank region of Palestine (29–31). An estimated 105,000 refugee adolescents reside in Palestine (32); however, little is known about the relationship between refugee status and blood pressure among Palestinian adolescents. This study examined the prevalence of prehypertension and hypertension among Palestinian adolescent refugees and non-refugees residing in the West Bank, Palestine, and assessed the effect of refugee status and sex on prehypertension and hypertension. Specifically, we hypothesized that the prevalence of prehypertension and hypertension would vary by (1) refugee status and (2) sex.

## Methods

The data supporting the findings of this study are available from the corresponding author upon reasonable request and with appropriate approvals, given the sensitive nature of the data collected.

### Study design

A cross-sectional study was conducted from 1 November 2022 to 10 January 10 2023 to explore the prevalence of prehypertension and hypertension among Palestinian adolescent refugee populations (refugee) compared to their non-refugee (non-refugee) counterparts residing in the West Bank, Palestine.

### Setting, sampling, and selection of participants

A stratified random sample of 706 Palestinian adolescents [377 refugee, 329 non-refugees], aged 13 to 17 years and residing in refugee camps, cities, and villages across four regions of the West Bank (i.e., Ramallah, Bethlehem, Nablus, and Hebron), was recruited. The four regions were selected based on geographical location, population density, and the presence of refugee camps. Sampling occurred in two stages: Palestinian Ministry of Education (PMOE) government schools and refugee camp community centers connected to United Nations Relief and Works Agency (UNRWA) schools were randomly selected across the regions using the lists provided by the PMOE and refugee camp community directors. Once schools and local community centers agreed to participate, a list of adolescents in grades 8 to 11 from each selected school and community center was provided to the investigator, who then randomly selected participants to recruit for the study. The inclusion criteria were as follows: adolescents aged 13–17 years attending government or UNRWA schools. Most Palestinian adolescents attend either UNRWA or PMOE government schools in the West Bank, where free education is offered. UNRWA schools in the West Bank were specifically established to serve refugee adolescents (28).

An information pamphlet providing details about the study and a consent form, both in Arabic, were sent home with adolescents for their caregivers (i.e., parent/legal guardian) to review alongside their adolescents who expressed interest in enrolling in the study. Caregivers’ and adolescents’ consent was obtained prior to enrollment in the study. Data collection was conducted at local community centers after school hours in each specified region.

### Ethical considerations

Ethical approval was granted by the Institutional Review Boards of the University of California, San Diego, An-Najah National University, and San Diego State University. Approvals from the Palestinian Ministry of Education and the Refugee Camp Community Board were obtained prior to study initiation. Participants were compensated $15 for their time and participation.

### Sample size

The sample was selected to detect differences in the prevalence of prehypertension and hypertension between Palestinian adolescent refugees and non-refugees, with a 95% confidence interval and a precision of ±5%. A sample size of 692 participants was originally calculated [379 refugees and 313 non-refugees]. This calculation was based on the assumption that adolescent Palestinian refugees have at least a 10% higher prevalence of elevated blood pressure and hypertension (i.e., 36% CI 31 to 41%) compared to their non-refugee counterparts (i.e., 26% CI 21 to 31%). This sample size aligns with previous research focused on elevated blood pressure among Palestinian adolescent populations (26, 27, 29, 33). Oversampling (i.e., allocation ratio = 1.12) among the refugee population strata was conducted to account for the refugee status exposure among adolescents, given that both refugee and non-refugee adolescents are affected by occupation. G-Power 3.1.9.4 was used for power calculations.

### Survey

The participants were asked to complete socio-demographic surveys that collected information regarding sex (male or female), age (13–17 years), school grade level (8–11), caregiver occupation (employed or unemployed), place of residence (refugee camp, city, village), family history of hypertension (yes or no), physical activity level (0 to 1 time a week, 2–3 times a week, and 4 or more times a week) (34), refugee status (registered refugee or non-refugee), and school type (UNRWA school or PMOE government school).

### Anthropometrics

The participants were asked to undergo three consecutive measurements: body weight (kg), height (cm), and body fat percentage (%). The height of the participants was measured using a measuring tape and, along with their sex, programmed into the body composition scale prior to obtaining weight and body fat percentage. The participants were asked to step onto the body composition scale without shoes, and the average weight (kg) and body fat percentage were recorded.

### Blood pressure

Trained research staff recorded three consecutive blood pressure readings using a validated blood pressure monitor with an appropriately sized cuff placed on the participant’s right upper arm. Measurements were taken with the participant seated, allowing a minimum interval of 3 min between each reading (14). The adolescents were asked to refrain from caffeine intake for at least 4 h prior to the measurement. The trained staff also instructed the adolescents to keep their legs uncrossed and feet on the floor during the blood pressure measurement. The blood pressure measurements were recorded as continuous measures. All three systolic (SBP) and diastolic (DBP) blood pressure readings were used to obtain the mean SBP and DBP values. Based on the average of the continuous blood pressure values, the participants were categorized into one of three groups: Normal, prehypertensive, or hypertensive. The blood pressure categories were defined using the American Family Physician guidelines for adolescents aged 13 years and older as follows: normotensive (i.e., <120/<80 mmHg), prehypertensive (i.e., 121–129/81-89 mmHg), and hypertensive (i.e., ≥130/≥80 mmHg) (14).

### Data analysis

All analyses were conducted using the R statistical software (31). All tests of significance were two-sided and had an alpha value of < 0.05. Descriptive statistics included means and standard deviations for all continuous variables and percentages for categorical variables by refugee status. Bivariate analyses using *t*-tests and χ2 tests for continuous and categorical variables, respectively, were conducted to compare participant characteristics by refugee status. To assess the relationship between refugee status and the blood pressure categories (i.e., normal, prehypertension, and hypertension), ordinal regression models were performed using cumulative link models with a logit link and flexible thresholds. Normal blood pressure (e.g., ≤ 120/80) was used as the reference category against which the other ordinal blood pressure categories were compared (prehypertension and hypertension). The cumulative link models explained the odds of participants being classified into higher blood pressure categories (prehypertension to hypertension) relative to the normal blood pressure category, with refugee status as the primary predictor. Covariates in the cumulative link models included age (continuous) and refugee status (binary: refugee vs. non-refugee), where model one included refugee status as the only predictor, while model two adjusted for age. The proportional odds assumption for both models was tested using a nominal test, and a likelihood ratio test was used to assess whether the inclusion of age improved model fit. Furthermore, interaction terms were included to assess whether the relationship between refugee status and outcomes differed by sex (i.e., binary: male vs. female), while adjusting for age (continuous). Odds ratios and 95% confidence intervals were used to quantify the strength and direction of the association between each predictor in its respective model and the prevalence of prehypertension and hypertension.

## Results

### General characteristics of the study participants

A total of 706 adolescents aged 13 to 17 years (Table 1) completed the study, and 695 adolescents had valid blood pressure data based on three repeated readings (98.4%). Among these adolescents, there was a significant difference in age between the refugee and non-refugee groups (*p* < 0.001) (Table 1).

**Table 1:** Participant demographics and characteristics by refugee status.

Characteristic	Non-refugee adolescents	Refugee adolescents	*p*-value	Number of participants missing data
Sample size (*n*)	329	377		
Age [years *n* (%)]			<0.001	2
13	81 (24.7)	164 (43.6)		
14	65 (19.8)	79 (21.0)		
15	88 (26.8)	67 (17.8)		
16	50 (15.2)	41 (10.9)		
17	44 (13.4)	25 (6.6)		
Sex [*n* (%)]			0.937	0
Female	131 (39.9)	148 (39.4)		
Male	198 (60.1)	229(60.6)		
Residence [*n* (%)]			<0.001	5
City	56 (17.1)	18 (4.8)		
Village	256 (78.3)	15 (4.0)		
Refugee Camp	15 (4.6)	343 (91.2)		
Monthly Family Income (*n* (%))			<0.001	118
Less than $300	35 (11.4)	106 (37.9)		
$301–650	62 (20.1)	88 (31.4)		
$651 or more	211 (68.5)	86 (30.7)		
Family History of High Blood Pressure [*n* (%)]	124 (37.7)	132 (35)	0.923	40
Mean Body Fat [%(SD)]	25 (9)	24 (10)	0.251	12
Weekly Physical Activity Status [*n* (%)]			0.159	20
0 to 1 Times	140 (43.6)	146 (40.0)		
2–3 Times	113 (35.2)	154 (42.2)		
4 or more times	68 (21.2)	65 (17.8)		
Heart rate [mean (SD)]	86.12 (11.99)	87.28 (13.09)	0.222	4
Blood pressure category [*n* (%)]			0.357	0
Normotensive	186 (57.1)	228 (61.8)		
Prehypertension	88 (27.0)	94 (25.5)		
Hypertension	52 (16.0)	47 (12.7)		

**Figure 1:**
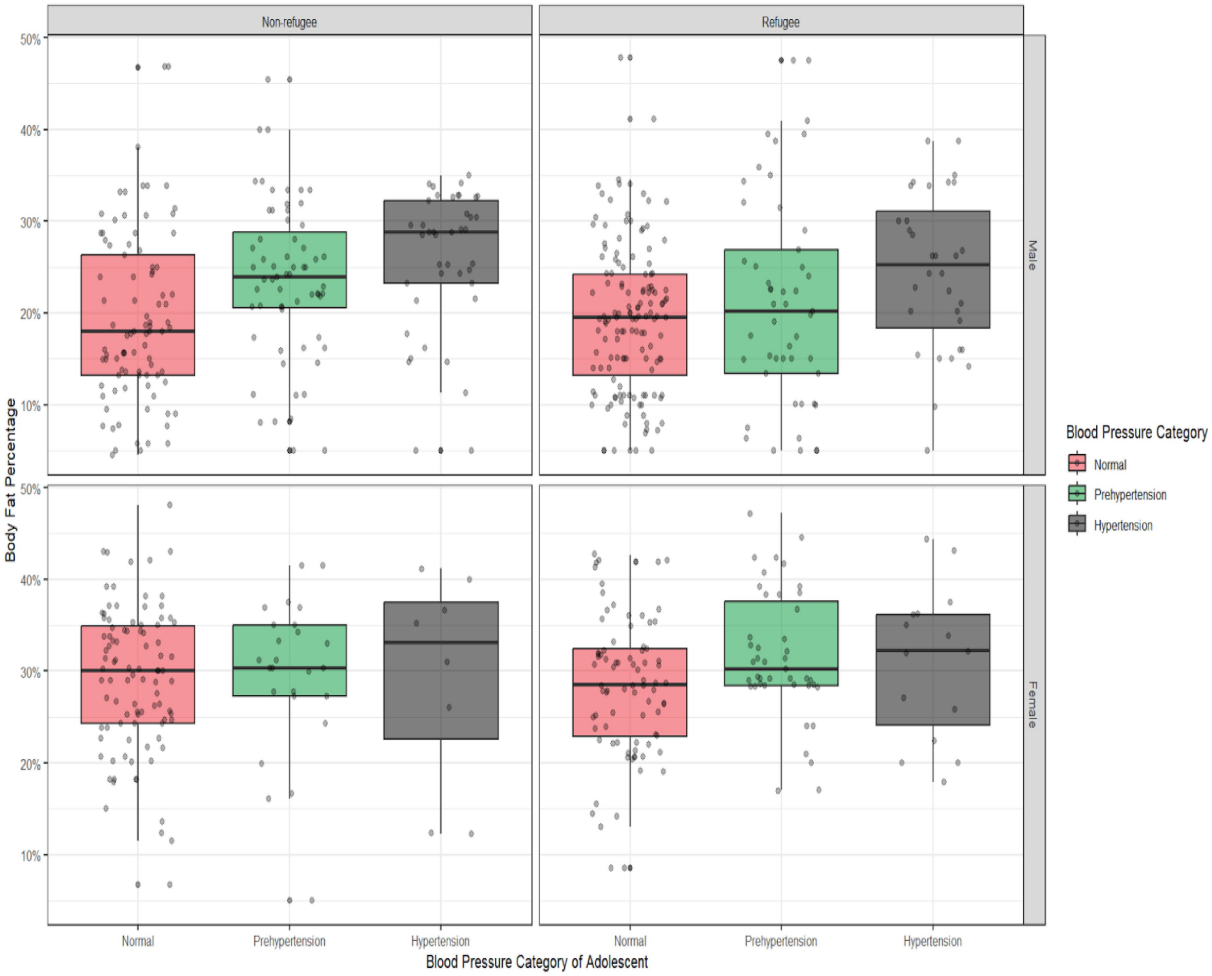
Body fat percentage by blood pressure categories among the Palestinian adolescents residing in the West Bank, Palestine, stratified by refugee status and sex. It shows the distribution of body fat percentage across the blood pressure categories (normal, prehypertension, and hypertension), stratified by sex and refugee status. The box plots represent the median, interquartile range, and outliers of body fat percentage within each category. Comparisons were made between the sex and refugee status subgroups to assess variations in body fat percentage.

In total, 10.5% of the participants resided in a city, 38.4% in villages, and 50.7% in refugee camps. The refugee adolescents reported lower monthly family incomes—less than $300 a month (38%)—compared to the non-refugee adolescents (11.4%) (*p* < 0.001) (Table 1). The majority of the adolescents reported exercising at least 2 or more times a week (60% refugee adolescents and 56.4% non-refugee adolescents). Overall, 36% of the adolescent sample reported a family history of hypertension, with similar rates among the refugees (32.1%) and non-refugees (32.8%). The distribution of body fat percentage was similar between the refugees [body fat percentage (SD), 24% (9)] and their non-refugee counterparts [body fat percentage (SD), 25% (10)] (Table 1 and Figure 1).

### Prevalence of prehypertension and hypertension by refugee status and sex

**Figure 2:**
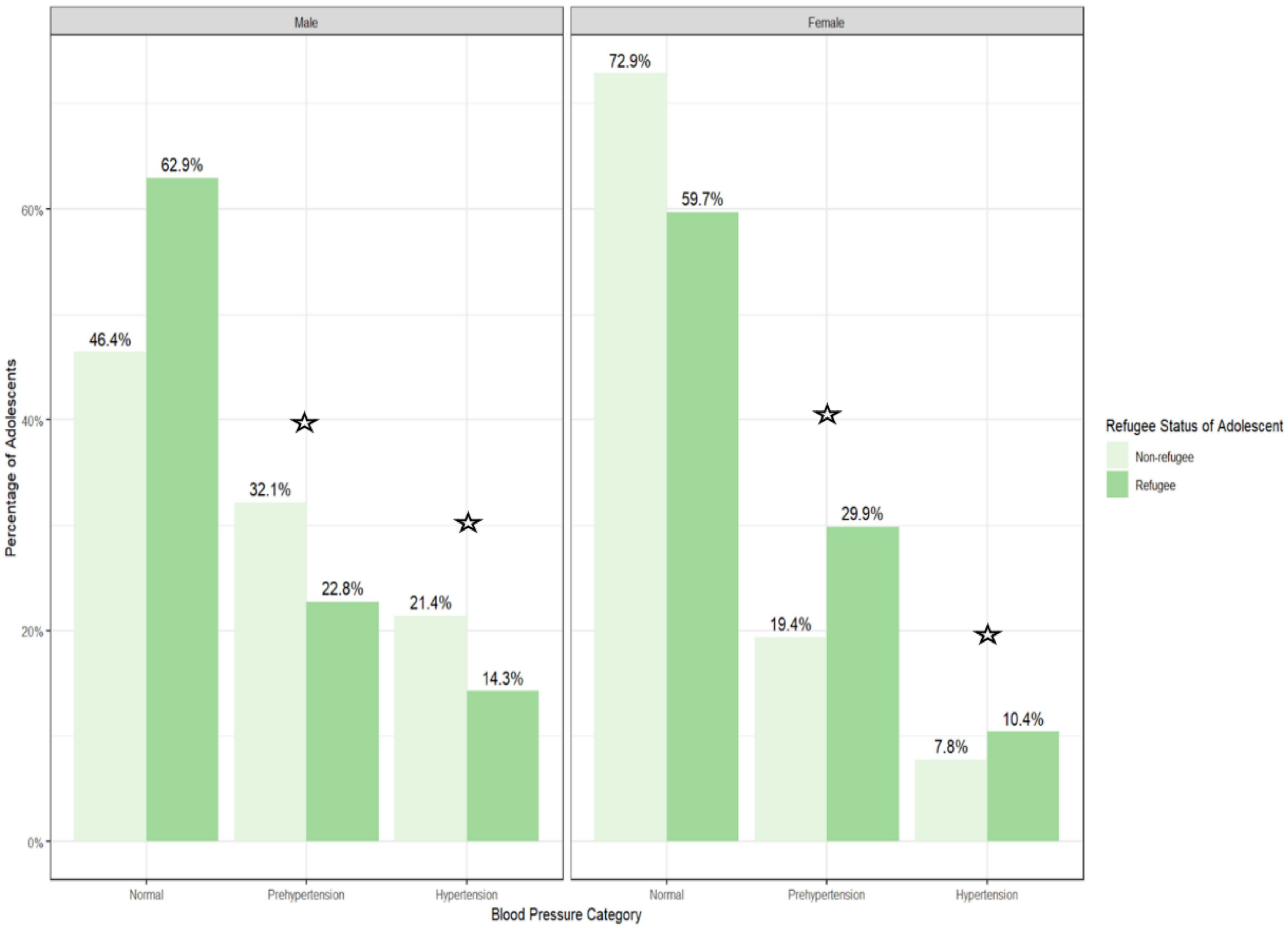
Prehypertension and hypertension prevalence among the palestinian adolescents residing in the West Bank, Palestine, by refugee status and sex. 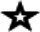 *p* < 0.05. The prevalence of blood pressure categories (normal, prehypertension, and hypertension) among the refugee and non-refugee Palestinian adolescents, stratified by sex. The bar heights represent the percentage of adolescents in each subgroup within each blood pressure category. Data are derived from a sample of 706 adolescents.

The overall prevalence of prehypertension and hypertension for the total sample was 26 and 14%, respectively (Table 1). The non-refugee adolescents in the sample displayed a higher prevalence of prehypertension [non-refugees= 27.0%, refugees= 25.5%] and hypertension [non-refugees=16.0%, refugees= 12.7%] compared to refugee adolescents (Table 1). As shown in Figure 2, overall, the male adolescents had a higher percentage of prehypertension [total male individuals = 54.9% (refugees = 22.8%, non-refugees = 32.1%)] and hypertension [total male individuals = 35.7% (refugees = 14.3% and non-refugees = 21.4%)] compared to the female adolescents, who had lower rates of prehypertension [total female individuals = 49.3% (refugees = 29.9%, non-refugees = 19.4%)] and hypertension [total female individuals = 18.2% (refugees = 10.4%, non-refugees = 7.8%)] (*p* < 0.0001). The female refugees in the sample had a higher prevalence of prehypertension and hypertension (29.9, 19.4%) compared to the female non-refugee adolescents (10.4, 7.8%) (Figure 2). In contrast, a higher prevalence of prehypertension and hypertension was observed among the male non-refugee adolescents (32.1, 21.4%) compared to the male refugee adolescents (22.8, 14.3%) (Figure 2).

Overall, there was no association between refugee status and the hypertension categories in the unadjusted models (Table 2) or models adjusted for age (Table 2). In addition, the models with and without the assumption of proportional odds were compared and showed no significant difference (χ^2^ = 0.11, *p* = 0.73), suggesting that the models did not violate the proportional odds assumptions (35). Furthermore, as presented in Figure 2 and Table 2, there was a significant association between refugee status, prehypertension, and hypertension by sex after adjusting for age, with higher odds of prehypertension and hypertension in the female refugees compared to the female non-refugees and lower odds of prehypertension and hypertension in the male refugees compared to the male non-refugees.

**Table 2:** Association between refugee status and high blood pressure and interaction analysis by sex.

Population	Odds ratio	95% Confidence interval
Association between refugee status and high blood pressure (i.e., prehypertension and hypertension)		
Refugee status (i.e., refugee)		
Crude odds ratio	0.81	0.60–1.09
Adjusted odds ratio (i.e., refugee x age)	0.88	0.65–1.20
Interaction model of refugee status, sex, and high blood pressure (i.e., prehypertension and hypertension)		
Female refugees	1.75	1.04–2.95
Male refugees	0.6	0.41–0.88

Interaction models were adjusted for age.

## Discussion

This cross-sectional study is the first study to document the burden of prehypertension and hypertension among a large sample of Palestinian refugee and non-refugee adolescents aged 13 to 17 years residing in diverse regions (i.e., North, South, East, and West) across the West Bank, Palestine. The unadjusted prevalence rates of prehypertension and hypertension were slightly higher in the non-refugee sample despite refugee status insignificance. However, sex differences emerged in the participant sample, where the female refugees exhibited higher odds of prehypertension and hypertension than the female non-refugees, while the male refugees had lower odds than the male non-refugees.

The prevalence rates of prehypertension and hypertension observed in this study among the Palestinian non-refugee adolescents were 7 and 5% lower, respectively, than those reported in two previous studies conducted among Palestinian non-refugee youth. Both studies were conducted on small samples of Palestinian youth aged 10 to 13 years and 16 to 18 years residing in the cities of Tubas, Jenin, and Nablus within the West Bank (30, 31). This finding may be attributed to increased exposure to socio-political and environment stress in cities as Tubas, Jenin, and Nablus, which face higher levels of violence due to the occupation compared to other areas in the West Bank (30, 31). It has been documented in the literature that stress increases the severity and prevalence of hypertension, particularly in marginalized populations (26, 32). Notably, the prevalence rates observed among our sample of Palestinian adolescent refugees align with the prevalence rates observed among a sample of Syrian refugee youth aged 10 to 17 years living under similar psychosocial conditions in refugee camps in Jordan (31).

Although no significant association was observed between refugee status and blood pressure in the overall sample, the female refugees had 75% higher odds of elevated blood pressure compared to the female non-refugees. Moreover, the male refugees had 40% lower odds of elevated blood pressure compared to the male non-refugees. Studies have shown anthropometric, metabolic, and cardiovascular differences between female and male individuals during adolescence (36). Female individuals have higher adrenal androgen concentrations and greater adiposity than male individuals during adolescence, which can account for differences in insulin sensitivity and may affect blood pressure levels (37). While comparative data on the relationship between sex differences and hypertension in refugee adolescents are limited, previous literature assessing diverse refugee groups and health outcomes such as post-traumatic stress disorder (PTSD) suggests that female refugees, compared to male refugees, may experience more profound physiological outcomes due to stress resulting from adversity (38). For example, a study assessing the impact of mental health challenges on hypertension among female African Refugees in Durban, South Africa, demonstrated higher odds of developing hypertension among female refugees who had at least one adverse childhood experience, denoting a relationship between sex and refugee experience (38). Ho et al. (36) showed similar findings, suggesting sex-specific consequences for pubertal and physical maturation and mental health trajectories among female refugee adolescents who have had threatening experiences of adversity compared to male refugee counterparts. Similarly, Ainamani et al. (39) found sex differences in exposure to different war-related traumatic events and the risk of developing PTSD. Specifically, Congolese female refugees in Uganda exhibited greater PTSD severity compared to male individuals when exposed to low or moderate levels of traumatizing events (39). Furthermore, Gettler et al. (20) found that male refugees were less likely to have elevated chronic stress markers, such as C-reactive protein levels, compared to female refugees. Elevation of such markers has been linked to an increased risk of CVD and mortality (40, 41). Such findings may be due to female refugees experiencing a greater psychosocial and physiological burden from chronic stress and trauma, resulting in poorer immune function (20). Furthermore, differences in exposure and vulnerability to violence, trauma, and abuse between female and male individuals may contribute to variations in well-being within the refugee population (20). Factors such as insulin sensitivity measurements and psychosocial contributors to chronic stress in diverse refugee groups by sex should be considered in future analyses to gain a better understanding of their impact on cardiovascular health.

### Implications

With 26 and 14% of the participants having prehypertension and hypertension, respectively, these data suggest that this sample of Palestinian adolescents has three times and two and a half times the global average prevalence rates of prehypertension and hypertension (22, 28). This denotes an alarming public health burden in this understudied population (42). Early identification of adolescents and children at high risk for adult hypertension and CVD, combined with early behavioral interventions, is essential for combating the global burden of hypertension (43).

Furthermore, these data suggest sex differences in hypertension levels among adolescents based on refugee status. Future research should assess diverse lifestyle and psychosocial factors related to refugee and non-refugee experiences in both male and female individuals to better understand their relationship to blood pressure before implementing behavioral interventions.

### Limitations

The limitations of this study should be considered when interpreting our prevalence estimates. The sample was imbalanced by age between the refugee and non-refugee groups, resulting in a larger sample of 13-year-old refugees compared to their non-refugee counterparts, which necessitated the use of age-adjusted models (44). Moreover, measurement errors during blood pressure and anthropometric assessments can arise from various factors that may influence the validity and reliability of the results. These errors include technique-related errors (e.g., improper placement of the blood pressure cuff), physiological errors (e.g., ‘white coat’ hypertension), and instrumentation errors (e.g., using tools such as bioelectrical impedance scales to measure body fat percentage). Such errors can contribute to inaccuracies in estimating the true prevalence. To minimize the risk of measurement errors, all research staff were trained on the study protocol and instructed to take three measurements of blood pressure, weight, height, and body fat percentage (14). Finally, sample weighting techniques were not applied to assess the likelihood of inclusion when providing population-based prevalence estimates due to geopolitical circumstances in the regions contributing to limited census data. While the study’s sampling technique ensured recruitment of adolescents across the entire West Bank region, cities such as Jenin, Tulkarem, East Jerusalem, and Gaza were inaccessible due to heightened occupation-related activity during the data collection period; therefore, the results cannot be generalized outside of the sampled region.

## Conclusion

High blood pressure in adolescents is a predictor of future risk for CVD and other adverse health outcomes. This study documents the high prevalence of elevated blood pressure among Palestinian adolescents residing in the West Bank. Prehypertension and hypertension are critical health issues among this sample of adolescents and need to be addressed in Palestine to reduce the risk of adult hypertension and CVD. Further research examining sex differences in blood pressure levels and inflammatory markers based on refugee status should be conducted before designing future interventions aimed at addressing cardiovascular health disparities among Palestinian adolescent refugees residing in Palestine. Future studies should adopt a life course perspective, examining the potential effects of refugee experiences on the development of CVD.

## Data Availability

The raw data supporting the conclusions of this article will be made available by the authors, without undue reservation.
